# Renal Artery Triplication: An Unusual Morphological Variant

**DOI:** 10.7759/cureus.59365

**Published:** 2024-04-30

**Authors:** George Tsakotos, Savvas Melissanidis, George Triantafyllou, Christos Koutserimpas, Maria Piagkou

**Affiliations:** 1 Department of Anatomy, School of Medicine, National and Kapodistrian University of Athens, Athens, GRC; 2 Department of Radiology, Asklipios MEdica, Veroia, GRC

**Keywords:** kidney transplantation, kidney, variation, triplication, renal artery

## Abstract

Renal vasculature depicts great morphological variability and clinical significance due to the great number of procedures performed on kidneys. The current imaging report presents a right-sided renal artery (RA) triplication and origin from the abdominal aorta (AA), which was incidentally identified during computed tomography angiography (CTA). The typical RA corresponded to the main hilar artery (MHA), the second RA corresponded to the superior polar artery (SPA), and the third RA corresponded to the inferior polar artery (IPA). RA triplication occurs in 0.9%-4.5% and depicts wide morphological variability. The current report corresponds to one superior polar, one inferior polar, and a main hilar renal artery, which represents a rare morphological type of RA triplication. Kidney transplantation surgery, endoscopic surgery, and renal angiography require adequate knowledge of RAs and their variants to avoid pitfalls and iatrogenic lesions from clinicians.

## Introduction

The typical renal artery (RA) originates from the abdominal aorta (AA) and divides into two main trunks that give off five to seven branches that enter the kidney's parenchyma while also supplying the renal pelvis, fatty capsule, upper part of the ureter, and adrenal gland [1]. The renal arterial system depicts high morphological variability, and it has been extensively studied. This variability can be attributed to the complex developmental background of the kidney and its vascular system. Specifically, the RA could be identified with different origin levels, variable branching patterns, or multiplication [1]. Except for anatomical interest, RA variants depict clinical implications due to the high number of procedures that are performed, such as kidney transplantation [1]. According to Lippert and Pabst [2], the typical RA is presented in 59%. The most frequent variant (13%) that can be observed is the polar artery originating from the RA main stem [2]. One accessory RA can be seen in 22%, while two or more accessory RAs (three or more RAs) in 4% [2]. Recently, multiple RAs with an extra-aortic origin coexisting with quadruple testicular veins were identified in a cadaveric sample [3]. Nevertheless, except for multiple RAs, multiple renal veins have also been studied [4], and their estimated pooled prevalence was 16.7% [5].

The current imaging report presents an unusual variant of a right-sided RA triplication. The embryological background and clinical significance of such variants are further discussed.

## Case presentation

**Figure 1:**
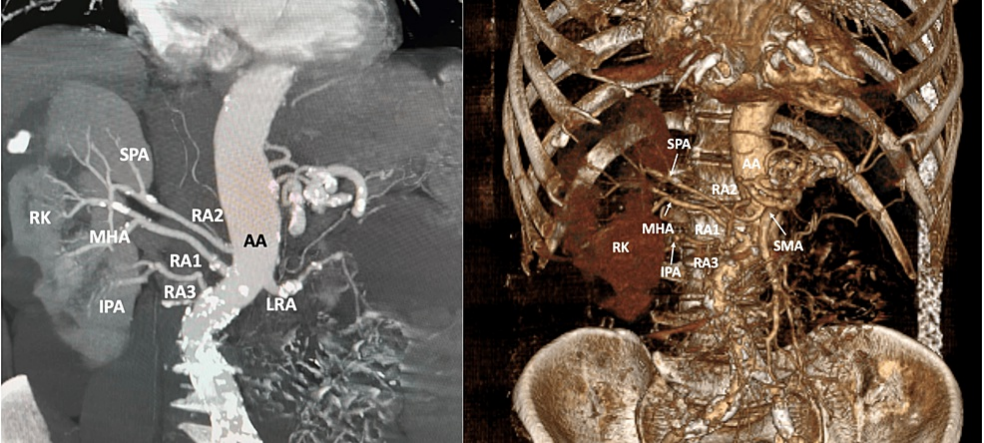
The right renal variant vasculature was identified in CTA before and after 3D reconstruction. Three renal arteries (RA1, RA2, and RA3) originated from the AA. After observing their course in the kidney, these arteries were characterized as the MHA, SPA, and IPA. CTA: computed tomography angiography, AA: abdominal aorta, MHA: main hilar artery, SPA: superior polar artery, IPA: inferior polar artery, RK: right kidney, SMA: superior mesenteric artery, LRA: left renal artery

The 71-year-old male patient proceeded to the Radiological Department for evaluation of an AA aneurysm that was identified after ultrasound. The patient underwent computed tomography angiography (CTA) for the evaluation of the aneurysm, while an interesting incidentally identified variant was observed. On the right side, an RA triplication was identified. The typical RA, characterized as the main hilar artery (MHA), originated from the AA (at the same level as the superior mesenteric artery) and is 53.9 mm in length. The second RA, the superior polar artery (SPA), was seen arising from the AA (11.7 mm superiorly to the main RA) and supplied the upper pole of the kidney, with a 70.8 mm length. The third RA, the inferior polar artery (IPA), was seen arising from the AA (11.5 mm inferiorly to the main) and supplied the lower pole of the kidney, with a 37.6 mm length, due to the AA tortuous course. The three RAs were branching into two trunks before entering the kidney's parenchyma (Figure 1).

## Discussion

Embryological development of renal vasculature

On an 18 mm fetus, the developing mesonephros, metanephros, adrenal glands, and gonads are vascularized from 18 to 20 branches from the AA. The middle group (3rd-5th arteries) gives rise to the adult RAs. Alteration to this procedure would lead to the persistence of more than one artery. Nevertheless, arterial variants can be formatted during the ascending of the mesonephros from the pelvic cavity to the lumbar region. This process is accompanied by changes in the vasculature; while the kidneys ascend, RAs arise at a different AA level. Hence, accessory RAs can represent persistent mesonephric arteries. Interestingly, analysis of the occurrence of accessory RAs showed that they can be identified more frequently in fetuses than in adults due to the ongoing process of kidney formation in fetuses [6].

Terminology and morphological variability of the renal artery (RA)

Although the renal vasculature has been extensively studied [2,3,7-12], its variant nomenclature remains controversial. The terminological problem occurs in the definition of "accessory" vessels. Different terms have been used throughout literature: "accessory," "multiple," "aberrant," "plural," or "supernumerary." According to Kachlik et al. [13], an accessory vessel (vas accessorium) is defined as a vessel supplying the same area as the proper one, branching from the proper source vessel or a neighbor one, while an aberrant vessel (vas aberrans) is defined as the vessel supplying the same area as the proper one but branching from another source vessel or a separate network. Hence, the terms "multiple," "plural," and "supernumerary" are different words to express the "accessory" vessel, while the "aberrant" vessel corresponds to a different entity.

In the current case, three arteries originate from the AA and were found to supply the right kidney. Hence, the current case report could be characterized as triple RAs. However, in the current report, is this definition precise? Due to the CTA that was performed, we had further knowledge of each artery, such as the area that they were supplying. The typical RA corresponds to the vessel that enters the kidney parenchyma, at the hilum, and gives off all the branches of the organ. Thus, all the arteries are segmental end-arteries of this vessel. In the current case, only one artery corresponded to the typical RA anatomy, while the other two supplied the superior and inferior pole of the kidney. These two variant arteries, which were identified as originating from the AA, should be branches of the main RA. Hence, to the best of our knowledge, the current case corresponds to three arteries supplying the right kidney: the main RA (MHA) and two ectopic branches (SPA and IPA). Lippert and Pabst [2] have systematically studied the RAs. According to them, two RAs can be identified in 22%, with different patterns such as two RAs running to the hilum (10%), one RA to the hilum and one RA to the upper pole (7%), and one RA to the hilum and one to the lower pole (5%). Three RAs are rarer (4%) and can be observed as follows: two hilar arteries, one lower polar (1%), and one hilar, one upper polar, and one lower polar artery (<1%). The last type corresponds to the current case and represents a rarer variant of three RAs.

Cases et al. [12] studied RAs in cadaveric and radiologic specimens and performed a literature review to systematically classify RA variants. According to their study, a total of five patterns were identified, according to the number of RAs supplying the kidney (pattern I: one artery, pattern II: two arteries, etc.). However, they mentioned that arterial patterns can be found in different morphological types. Three RAs were identified in 2.3%, without further analysis for the different morphology of the three RAs [12]. The current case report was identified to a Greek male patient; so, it is important to mention similar variants identified in a Greek population. Natsis et al. [11] performed a cadaveric study on 103 Greek cadavers and studied the presence of "multiple" RAs. They identified one accessory RA in 9.2% of the kidneys, two accessory RAs in 1.5%, and three accessory RAs in 0.5% [11]. Papaloucas et al. [10] performed a CTA study on 215 Greek patients (430 kidneys), and they identified one accessory RA in 21.4% and two accessory arteries in 5.6% [10]. Except for an original study, Natsis et al. [10] performed a detailed literature review and identified the presence of three RAs (two "accessory" and one main) ranging between 0.9% and 4.5% [8,9], without further analysis of the different morphological types. It is worth mentioning that the lowest frequency (0.9%) occurred in the highest sample (1,710 kidneys) of the included studies. In their study, Ozkan et al. [8] reported nine triple right-sided RAs (1%) and six left-sided triple RAs (0.7%). Damaskos et al. [14] reported a surgical case of three RAs at a cadaveric donor during renal transplantation.

Clinical significance

Adequate knowledge of renal vasculature variants is of paramount importance, especially for interventionists, to reduce pitfalls and accidental intraoperative injuries, mostly for renal transplantation. Kidney transplantation with accessory arteries requires a more demanding approach and more time for vascular anastomoses, resulting in elongation of the warm ischemia time, a negative factor for the graft [14]. Except for graft risk, the recipients are at high risk of vascular complications and thrombosis [14]. Furthermore, there is an unproved association between accessory RAs and hypertension [11]. Nevertheless, urological complications can occur because accessory RAs and lower polar arteries commonly course anteriorly to the ureteropelvic junction and are considered an etiologic factor of hydronephrosis [11].

## Conclusions

The current case presents a quite rare triplication of RA variants, supplying the right kidney. Especially, the morphological type of these arteries (hilar, superior polar, and inferior polar) represents a rare morphological type of triple RAs. Knowledge of RA variants is important for clinicians, especially for renal transplantation surgery.
